# Time spent with HIV-RNA ≤ 200 copies/ml in a cohort of people with HIV during the U=U era

**DOI:** 10.1097/QAD.0000000000002825

**Published:** 2021-02-09

**Authors:** Giordano Madeddu, Andrea De Vito, Alessandro Cozzi-Lepri, Antonella Cingolani, Franco Maggiolo, Carlo Federico Perno, Roberta Gagliardini, Giulia Marchetti, Annalisa Saracino, Antonella d’Arminio Monforte, Andrea Antinori, Enrico Girardi

**Affiliations:** a Unit of Infectious Diseases, Department of Medical, Surgical and Experimental Sciences, University of Sassari, Sassari, Italy; b Centre for Clinical Research, Epidemiology, Modelling and Evaluation (CREME) Institute for Global Health UCL, London, UK; c Institute of Clinical Infectious Diseases, Catholic University of the Sacred Heart, Rome; d Division of Infectious Diseases, ASST Papa Giovanni XXIII, Bergamo; e Department of Laboratory Medicine, Unit of Microbiology and Immunology, IRCCS Children Hospital Bambino Gesù; f HIV/AIDS Clinical Unit, National Institute for Infectious Diseases Lazzaro Spallanzani IRCCS, Rome; g ASST Santi Paolo e Carlo, Department of Health Sciences, University of Milan, Clinic of Infectious and Tropical Diseases, Milan; h Clinic of Infectious Diseases, Department of Biomedical Sciences and Human Oncology, University of Bari “Aldo Moro”, Bari; i Clinical Epidemiology Unit, National Institute for Infectious Diseases Lazzaro Spallanzani IRCCS, Rome, Italy.

**Keywords:** combination antiretroviral therapy, HIV, transmission, U=U message

## Abstract

Zero risk of linked HIV transmission in serodiscordant couples when the HIV-infected partner had viral load less than 200 copies/ml (‘U status’) was found in observational studies. We aimed at estimating the proportion of time in which ‘U status’ was maintained and identifying factors associated with the risk of losing it.

**Design:**

Observational cohort study.

**Methods:**

We included participants in the ICONA cohort who had reached an established ‘U status’ (viral load ≤200 copies/ml for >6 months) as of December 2010. The outcome was the number of person-days of follow-up (PDFU) above a viral load greater than 200 copies/ml, relative to the total number of PDFU observed. A logistic regression model was used to identify factors independently associated with the risk of losing ‘U status’.

**Results:**

Eight thousand, two hundred and forty-one persons living with HIV were included in the analysis who contributed 2 670 888 PDFU. Of these, 1648 (20%) were women, 768 (9%) were people who inject drugs (PWID), and 2066 (25%) were foreign-born. The median of viral load measurements was 9 (IQR: 4–15). Overall, only 3.1% of PDFU were observed when viral load was above 200 copies/ml. The proportion of PDFU with viral load more than 200 copies/ml was higher than average in women (5.3%), unemployed (5.4%), PWID (4.7%), and in people with more than three previous virologic failures (6.3%). These variables were significant predictors of losing ‘U status’ in the multivariable logistic regression.

**Conclusion:**

Our results reinforce the validity of the U=U message in real-world setting. However, we identified subsets of our study population at higher risk of losing the ‘U status’ for whom additional efforts are needed.

## Introduction

Since the end of the 1980s, several studies have investigated the association between HIV replication, as measured by plasma HIV-RNA or p24 antigen, and the risk of sexual transmission of HIV [1–6]. In 1994, an Italian study demonstrated how patients treated with zidovudine had a reduced transmission rate to their female partners [7].

After introducing combination antiretroviral therapy (cART), several investigators hypothesized that treatments could decrease the risk of sexual HIV transmission and that a person with an undetectable HIV-RNA may not transmit the virus [8].

In 2008, the Swiss National AIDS Commission published a document stating that ‘an HIV-infected individual without additional sexually transmitted disease (STD) and on cART with completely suppressed viraemia is sexually noninfectious’ [9]. In a meta-analysis published in 2009, Attia *et al.* suggested that there was no direct evidence that the HIV sexual transmission risk in serodiscordant couples was zero. Therefore, the definition of a transmission threshold was questionable or needed to be lower than previously believed [10]. However, in this metanalysis, no case of sexual transmission was observed in people with an HIV-RNA less than 400 copies/ml while on cART, despite the fact that the confidence interval (CI) for the zero rate was wide.

The HIV Prevention Trials Network (HPTN) developed a randomized and controlled trial (HPTN052), enrolling 1750 serodiscordant couples. The purpose of this trial was to determine whether cART can prevent HIV sexual transmission in serodiscordant couples who started treatment immediately vs. couples who delayed treatment start. At the end of the follow-up, no HIV transmission was registered among couples, provided the HIV-positive partner had an undetectable (HIV-RNA <200 copies/ml) viral load. Although no cases of HIV sexual transmission was reported in HPTN052, the CI was still too large (0–0.26) to be used to support the ‘zero risk statement’ [11].

After HPTN052, three observational studies, Partner 1, Partner 2, and Opposites Attract showed no cases of HIV transmission among both heterosexuals and MSM serodiscordant couples [12–14]. In 2019, a systematic review including data on over 4000 couple-years of follow-up was analysed by Vernazza [15]. A combined HIV sexual transmission risk of 0.00 [confidence interval (CI) 0.00 – 0.07)] while the HIV-positive person was virally suppressed, was estimated.

In February 2016, after that Partner 1 results were first published, the Prevention Access Campaign launched the Undetectable=Untransmittable (U=U) slogan. Since then, more than 750 organizations worldwide, including the WHO, took part in the U=U campaign. Even if these data have been enthusiastically approved by the community and by most scientists, several others have expressed concerns, mainly related to the persistence of undetectable viral load over time. The frequency of viral load determination to be sure that the undetectability lasts over time is still debated.

Although the absence of risk of transmission in the presence of viral suppression is well established in clinical trials and observational studies, there is little information on how long a viral load 200 copies/ml or less can be maintained in persons under routine clinical care. The present study aims to estimate, in a target population of people with HIV (PWH) seen for care in Italy who achieved a stable ‘undetectable (U) status’, the proportion of time in which such status was maintained over follow-up and to identify factors associated with the risk of eventually losing the ‘U status’.

## Methods

The Icona Foundation Study is an Italian cohort of patients with HIV that followed the original ICONA study [16]. We included participants in the ICONA cohort who had achieved a stable ‘U status’ (viral load ≤200 copies/ml for >6 months); entry in the analysis was set at the time of the first of two consecutive viral load 200 copies/ml or less experienced on ART after 1 June 2010 (baseline) until 31 July 2019. All patients signed consent forms to participate in the Icona Foundation Study in their local participating clinical sites. The research study protocol has been approved by local institutional review boards.

**Fig. 1:**
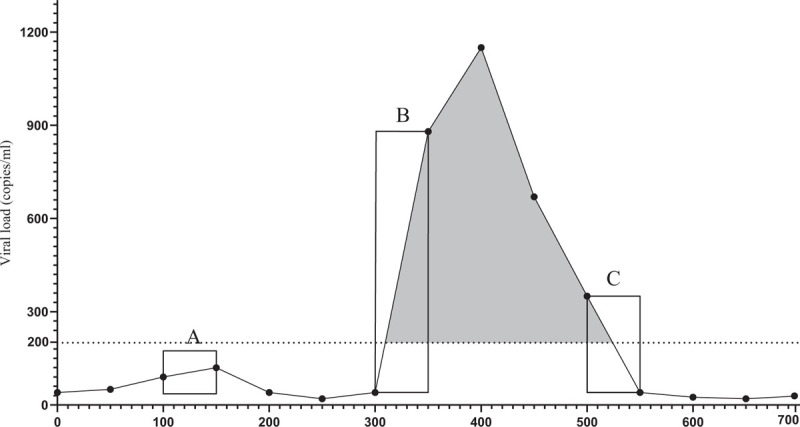
Estimating person days of follow-up with viral load greater than or 200 copies/ml or less for a hypothetical participant.

The number of person-days of follow-up (PDFU) spent above or below a threshold were calculated for individual participants using consecutive viral load pairs and the method proposed by Marks *et al.*[17] (Fig. 1). In detail, if both viral load measurements of a consecutive pair were more than 200 copies/ml, then all intervening PDFU were considered to have been greater than 200 copies/ml (grey area in Fig. 1). If both viral load measurements of a pair were 200 copies/ml or less, all intervening PDFU were considered 200 copies/ml or less (square A in Fig. 1). If the first viral load in the pair was 200 copies/ml or less and the second was above 200 copies/ml (square B), or vice versa (square C), we used a straight-line approximation to estimate viral loads between measurements.

If both viral load measurements of a pair were 200 copies/ml or less (square A), then all intervening person-days of follow-up (PDFU) were considered to have been 200 copies/ml or less. The grey area represents the PDFU greater than 200 copies/ml. If both measurements of a pair were greater than 200 copies/ml viral load, then all intervening PDFU were considered to have been greater than 200 copies/ml. If the first viral load in the pair was 200 copies/ml or less and the second was above 200 copies/ml (square B), or vice versa (square C), we used a straight-line approximation to estimate viral loads between measurements.

The primary outcome was then defined at the population level as the proportion of PDFU spent with a viral load above 200 copies/ml, relative to the total number of PDFU.

In addition, this same proportion was also used to define a binary endpoint for individual participants: losing the ‘U status’ over follow-up (yes/no). This was defined through the arbitrary cut-off of 10%, that is, if the proportion of PDFU for a person over the whole observation period was above10%, it was defined as having lost such a status (Fig. 1).

The main characteristics of the participants at baseline were compared between those who remained or lost the ‘U status’ using chi-square or Mann--Whitney *U* test, as appropriate. A multivariable logistic regression analysis was also performed to identify factors independently associated with the risk of losing the ‘U status’. All factors considered *a priori* to be significant predictors, based on the literature or other axiomatic knowledge, have been included in the multivariable model, regardless of the *P* value in the unadjusted comparison. Key exposure factors were defined as follows: a history of virological failure was a binary variable classified as ‘Yes’ if the participant had experienced before baseline at least 4 months with an HIV-RNA above 500 copies/ml while receiving ART, ‘No’ otherwise; hepatitis co-infection was defined based on the results of serology test; all clinical diagnoses of STDs before baseline was counted. Finally, we considered both the most recent CD4^+^ cell count prior to baseline (within 6 months of this date) and the CD4^+^ cell count nadir.

Alternative endpoints, less dependent on participants’ length of follow-up, were examined. In one of these analyses, losing the ‘U status’ was defined as experiencing at least 3 months with a viral load greater than 200 copies/ml.

We have also performed a sensitivity analysis after removing participants whose HIV-RNA monitoring was less frequent than twice a year, which is the average monitoring frequency by the protocol in the cohort.

## Results

Out of all PWH enrolled in the Icona cohort, 8241 participants were included in this analysis. Of these, 1648 (20%) were women, and the foreign-born were 2066 (25.1%).

Concerning the modality for acquiring HIV infection, 768 (9.3%) were people who inject drugs (PWID), 3786 (45.9%) MSM, and 3176 (38.5%) heterosexuals.

Four-hundred and six (4.9%) participants have had AIDS-defining events before baseline. The median age at baseline was 39 [interquartile range (IQR) 31–47] years, with a median of CD4^+^ cell count of 545 (IQR 400–722) cells/μl. The majority of participants (7488; 90.9%) have never had a history of virological failure before baseline. However, 429 (5.2%) PWH had previously experienced a number of virological failures between one and three, and 324 (3.9%) had more than three virological failures.

The median of viral load measurements over the study period was nine (IQR 4–15), and the median time with viral load above 200 copies/ml was 47.3 days (IQR 46.3–47.9). Thus, HIV-RNA was collected at least twice a year (average 2.5 times per year), which is the monitoring frequency by the cohort protocol. The main baseline patients’ characteristics have been summarized in Table 1.

**Table 1:** Characteristics of 8241 patients meeting the definition of U status (i.e. a HIV-RNA ≤200 copies/ml) in the ICONA cohort.

	Total	90% or less time	More than 90% time	*P* value^b^
	(*N* = 8241)	(*N* = 617)	(*N* = 7624)	
Female [*n* (%)]	1648 (20.0%)	212 (34.4%)	1436 (18.8%)	<0.001
Age^a^ (years)	39 (31--47)	39 (32--47)	39 (30--47)	0.435
Mode of HIV transmission [*n* (%)]				<0.001
PWID	768 (9.3%)	115 (18.6%)	653 (8.6%)	
MSM	3786 (45.9%)	173 (28.0%)	3613 (47.4%)	
Heterosexual contacts	3176 (38.5%)	289 (46.8%)	2887 (37.9%)	
Foreign-born [*n* (%)]	2066 (25.1%)	190 (30.8%)	1876 (24.6%)	<0.001
Education [*n* (%)]				<0.001
Primary school	426 (5.2%)	59 (9.6%)	367 (4.8%)	
Secondary school	1629 (19.8%)	164 (26.6%)	1465 (19.2%)	
College	2579 (31.3%)	161 (26.1%)	2418 (31.7%)	
University	1019 (12.4%)	45 (7.3%)	974 (12.8%)	
Employment [*n* (%)]				<0.001
Unemployed	952 (14.0%)	123 (23.5%)	829 (13.2%)	
Employed	3488 (51.4%)	226 (43.2%)	3262 (52.0%)	
Self-employed	1187 (17.5%)	69 (13.2%)	1118 (17.8%)	
HbsAg+ [*n* (%)]	108 (1.3%)	8 (1.3%)	100 (1.3%)	0.716
HCVAb+ [*n* (%)]	905 (11.0%)	128 (20.7%)	777 (10.2%)	<0.001
Diabetes [*n* (%)]	231 (2.8%)	26 (4.2%)	205 (2.7%)	0.027
Smoking [*n* (%)]	2939 (35.7%)	253 (41.0%)	2686 (35.2%)	0.004
CVD diagnosis [*n* (%)]	87 (1.1%)	13 (2.1%)	74 (1.0%)	0.008
Prior STDs	1862 (22.6%)	144 (23.3%)	1725 (22.6%)	<0.001
AIDS diagnosis [*n* (%)]	1009 (12.2%)	110 (17.8%)	899 (11.8%)	<0.001
CD4^+^ cell count^a^ (cells/μl)	532 (356--730)	507 (302--698)	534 (361--733)	<0.001
CD4^+^ cell count nadir^a^ (cells/μl)	300 (162--436)	264 (119--403)	302 (166--438)	<0.001
CD8^+^ cell count^a^ (cells/μl)	878 (633--1198)	900 (661--1244)	874 (632--1195)	0.111
Peak viral load in follow-up^a^ (log10 copies/ml)	4.52 (3.67--5.12)	4.81 (4.16--5.35)	4.49 (3.63--5.11)	<0.001
CD4^+^ cell count ≤200 (cells/μl) [*n* (%)]	824 (10.0%)	94 (15.2%)	730 (9.6%)	<0.001
Time from HIV diagnosis^a^ months	15 (7--65)	31 (9--115)	14 (7--61)	<0.001
Follow-up time^a^ (months)	45 (21--78)	58 (30--82)	44 (20--78)	<0.001
No previous VF [*n* (%)]				<0.001
1–3	429 (5.2%)	54 (8.8%)	375 (4.9%)	
3+	324 (3.9%)	65 (10.5%)	259 (3.4%)	

ART, antiretroviral therapy; CVD, cardiovascular disease; PWID, people who inject drug; STDs, sexual transmitted diseases; VF, virologic failure.

a Median (IQR).

b Chi-square or Mann--Whitney *U* test as appropriate.

Overall, 617 participants (7.5%) spent less than 90% of PDFU with a viral load 200 copies/ml or less and were classified as losing their initial ‘U status’ over time. Approximately 65% of participants showed less than two values above 200 copies/ml over follow-up, and 30% of the participants experienced a single sustained episode, and approximately an additional 7% experienced two or more episodes (Supplemental Figure 1).

At univariable analysis (Table 1), when comparing PWH who did not retain the ‘U status’ over time (*n* = 617) with those who did (*n* = 7624), the first were more frequently female individuals (*P* < 0.001), PWID (*P* < 0.001), and foreign-born (*P* < 0.001). They were also less likely to have achieved a college or university degree (*P* < 0.001) and were more frequently unemployed (*P* < 0.001).

Regarding HIV-related characteristics, PWH who had not retained the ‘U status’ overtime had also been more frequently previously diagnosed with AIDS (*P* = 0.008), less likely having a co-infection (*P* < 0.001) and showed lower current and nadir CD4^+^ cell count (*P* < 0.001), higher peak median viral load (*P* < 0.001). Furthermore, they showed a longer time from HIV diagnosis to baseline (*P* = 0.002), had been followed up for longer, and were more likely to have previously experienced one or more episodes of virological failure (*P* < 0.001).

**Fig. 2:**
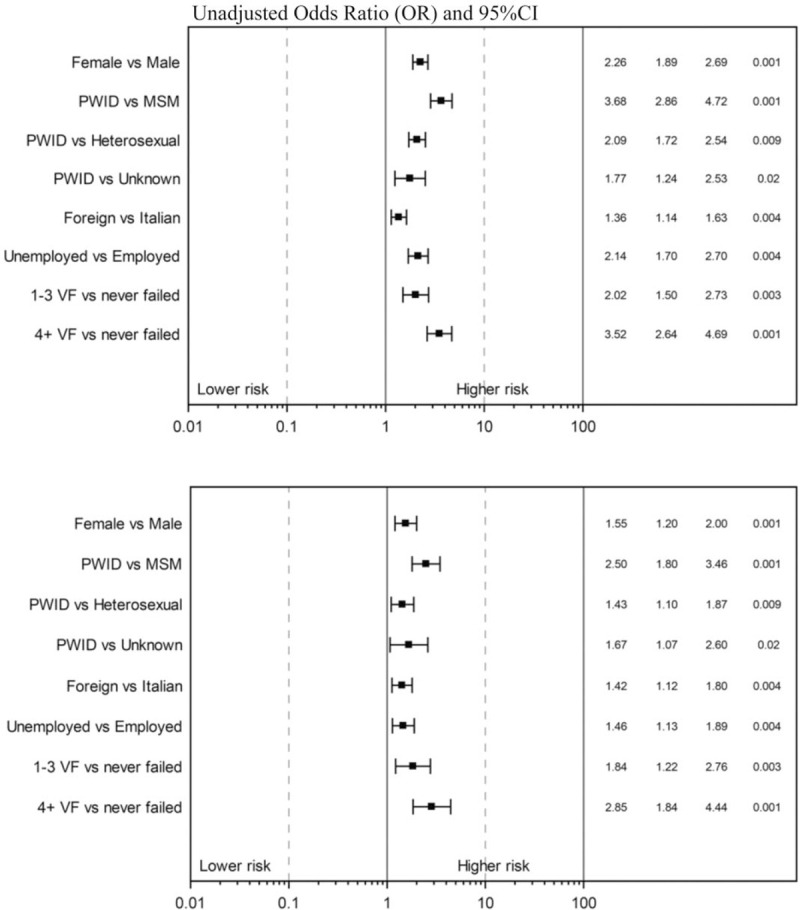
Forrest plot of multivariable logistic regression estimates of factors associated with losing ‘U status’.

In order to identify factors independently associated with the outcome, a multivariable logistic regression has been performed. In this unadjusted analysis, female sex, being PWID, foreign-born, low educational level, and unemployed, were independent predictors of losing ‘U status’. Having previously experienced at least one virological failure episode was also associated with a significantly higher risk of losing ‘U status’. The same variables were also confirmed as significant predictors of losing ‘U status’ after controlling for age, AIDS diagnosis, HBsAg/HCV status, duration of ART, anchor drug used, geographical region, diabetes, smoking, use of statins/lowering blood pressure drugs, glucose, and prior STDs. Unadjusted and adjusted odds ratios of losing ‘U status’ from fitting the logistic regression model are shown in Fig. 2. The adjusted odds ratio (aOR) for the main independent predictors were 1.55 [95% confidence interval (CI) 1.20–2.00] comparing women vs. men, 2.50 (95% CI 1.80–3.46), and 1.43 (95% CI 1.10–1.87) comparing PWID vs. MSM and heterosexuals, respectively. Being foreign-born had aOR of 1.42 (95% CI 1.12–1.80) vs. Italians, and 1.46 (95% CI 1.13–1.89) comparing unemployed vs. employed. Previous virological failure (>3) had an aOR of 2.85 (95% CI 1.84–4.44). When we used the alternative endpoint of at least 3 months with a viral load above 200, results were similar (Supplemental Table 1S). The magnitude of these associations was similar after restricting the analysis to participants with HIV-RNA monitoring of at least two measures/year (Supplemental Figure 2).

Multivariable model includes all variables selected by backward selection that were retained with a *P* value less than 0.3 level. Also adjusted for age, AIDS diagnosis, HBsAg/hepatitis C virus (HCV) status, duration of antiretroviral therapy (ART), anchor drug used, geographical region, diabetes, smoking, use of statins/lowering blood pressure drugs, glucose, and prior sexually transmitted diseases. PWID, people who inject drugs; VF: virological failure.

**Fig. 3:**
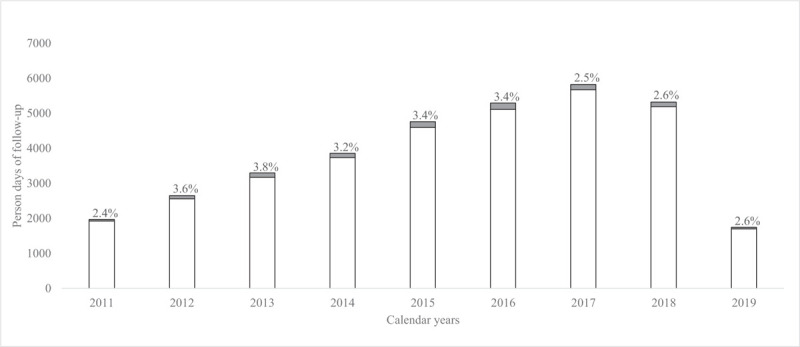
Person-days of follow-up with viral load greater than 200 copies/ml by calendar year of follow-up.

When using the PDFU as the statistical unit instead of the number of PWH, a total of 12 670 888 PDFU were counted over the entire observation period (2011–2019). Of these, 96.9% of PDFU were spent with a viral load 200 copies/ml or less. Thus, only the remaining 3.1% of PDFU were observed when viral load was above 200 copies/ml. The highest proportion of time spent with a viral load above 200 copies/ml was observed in 2013, with a progressive decrease in the following years, especially after 2016 (Fig. 3). Of note, in a sensitivity analysis restricted to people with HIV-RNA monitoring of at least two measures/year, the percentage of PDFU above 200 copies/ml was even lower at 2.5% (650/25 663).

The grey area represents the PDFU with HIV-RNA greater than 200 copies/ml. PDFU, Person-days of follow-up.

The proportion of PDFU with viral load greater than 200 copies/ml was significantly higher than average in women (5.3%), foreign-born (5.4%), unemployed (5.4%), PWID (4.7%), and in people with more than three previous virological failures (6.3%). There was no evidence of a difference in proportions when comparing patients with ages between 18–45 and at least 46 years (Table 2).

**Table 2:** Distribution of person-days of follow-up according to time spent in viral load categories and risk factors.

	HIV-RNA category (copies/ml)			
Variables	≤200	>200	% >200	*P* value
Gender				<0.001
Male	26856	671.8	2.4	
Female	7034	393.2	5.3	
Age				0.091
46+	12345	361.0	2.8	
18–45	21371	698.5	3.2	
Mode of HIV transmission				<0.001
MSM	14529	259.4	1.8	
Heterosexuals	13629	480.1	3.4	
PWID	1972	97.9	4.7	
Foreign-born				<0.001
No	28835	777.2	2.6	
Yes	5055	287.7	5.4	
Employment				<0.001
Self-employed	5306	126.5	2.3	
Employed	15847	443.6	2.7	
Unemployed	3449	198.1	5.4	
No. previous VF				<0.001
0	29262	809.6	2.7	
1–3	2644	122.7	4.4	
>3	1985	132.6	6.3	

PWID, people who inject drug; VF, virologic failure.

## Discussion

The Prevention Access Campaign (PAC) launched the slogan ‘Undetectable=Untransmittable’ or ‘U=U’ in 2016. PAC officially started this U=U prevention campaign after the publication of two studies, HPTN052 and Partner 1 [11,12]. The subsequent publication of the other two studies, Partner 2, and Opposites Attract studies [13–14], encouraged even more people to support the U=U message campaign. In these four studies, no cases of sexual transmission were registered when the HIV-positive partner had an HIV viral load under 200 copies/ml.

However, in HPTN052 and Partner 1, the serodiscordant couples were both heterosexual and MSM. On the contrary, in Partner 2 and Opposites Attract, only MSM couples were included. In none of the four studies, data about PWID and foreign-born were available. Furthermore, information on whether participants have had an AIDS-defining disease or precedent virological failure were not recorded. Although there is no strong reason to think that results would have been different in different target populations, strictly speaking, the quantitative results seen in these studies do not apply to these different case-mix populations.

Our analysis using the data of a cohort of PWH seen for care in Italy evaluated, for the first time, the probability of retaining an HIV-RNA 200 copies/ml or less (the ‘U status’) over a long period during the U=U prevention campaign era.

We found that in our population of PWH, the ‘U status’ was maintained, on average, for 97% of the following 10 years of observation, and the proportion of PDFU spent in the ‘U status’ showed a trend for an increase in recent years. Although a causal link cannot be established and we cannot ascribe these results to the correct receipt of the U=U message because of very high rates of viral suppression, this data reassuringly suggests that U=U is an appropriate message to communicate to help decrease stigma and increase motivation to remain virally suppressed. Of note, although the risk of transmission was not evaluated in the analysis, our study population included MSM, heterosexual women, and men, as well as PWID and foreign-born, subpopulations, which were underrepresented in other studies.

Furthermore, when fitting a multivariable logistic regression analysis, female sex, being PWID, foreign-born, and unemployed, were all independently associated with the risk of losing ‘U status’.

Looking at each of these factors in detail, we found that women have lost the ‘U status’ more frequently than men (5.3 vs. 2.4%). The role of female sex as a risk factor associated with virological failure risk is debated in the literature [18]. A meta-analysis of RCT showed no difference in the probability of reaching a virological success by sex [19]. However, in a real-life observational cohort, after controlling for other variables, women resulted at increased risk of delayed ART initiation and therapy discontinuation, even if no differences in response rates to the first year of ART were shown [20].

Foreign nationality has already been associated with an increased risk of virological failure. Saracino *et al.*[21] in the same cohort analysed here, showed that the incidence rate of viral failure was higher in foreign-born (15.5 per 100 person-years) compared with Italian-born (8.9 per 100 person-years). More recently, Reyes-Urueña *et al.*[22] showed that foreign-born had a significantly lower proportion of patients under follow-up with viral suppression compared with Spanish-born (82.9 vs. 87.2%). Furthermore, PWH originating from Latin America or the Caribbean showed a significantly increased risk [odds ratio (OR) 6.59; 95% CI 2.08–20.92] of virological failure compared with those born in the Netherlands in the Athena cohort [23].

The use of injection drugs was the most significant risk factor in losing ‘U status’ in our analysis. It has been previously associated with low adherence levels to cART, risk of treatment discontinuation, and virological failure. In the Swiss HIV cohort, Weber *et al.*[24] conducted a study on 6529 participants who were followed up for a total of 31 215 person-years. They showed that drug use lowered cART adherence, increased cART change rates and cART interruptions and that virological failure was more frequent among PWID. In the EuroSIDA cohort, PWID in East Central and Eastern Europe were around half as likely as MSM to have a suppressed viral load on ART (aOR 0.52; 95% CI 0.25–1.06) [25]. Furthermore, a longitudinal study conducted in the United States has shown that only 10.2% of PWID had sustained virologic suppression (HIV-RNA <400 copies/ml) during a median follow-up of 8.7 years [26].

PWH with poor education and those who were unemployed showed, in our analysis, a significant risk in losing the ‘U status’, compared, respectively, to PWH with advanced education and those employed. Many other studies demonstrated how these social determinants play a crucial role in response to antiretroviral therapy. D’Almeida *et al.*[27] aimed that the unemployed, compared with the employed, had a lower sustained virological suppression (aOR = 0.6). Saracino *et al.*[20] showed in 8023 PWH that the unemployed and people with lower education had a higher 1-year risk of first-line ART discontinuation. The COHERE study found that virological response was significantly related to the degree of education (67% in PWH without completed basic vs. 87% with tertiary education) [28]. Socioeconomic factors are seldom collected in HIV cohort studies, so this is one of our analysis strengths.

When considering virological and immunologic characteristics, losing ‘U status’ in our cohort was most frequently observed in PWH with a high peak viral load and in those with a CD4^+^ cell count 200 cells/μl or less. In a cohort study with 5766 PWH, Armenia *et al.*[29] demonstrated how people with a high viraemia had a high probability of experiencing a virological rebound with an HIV-RNA above 200 copies/ml. The role of CD4^+^ cell count as a predictor of virological failure is controversial. In the HPTN052 study, Eshleman *et al.*[30] found that PWH with a high CD4^+^ cell count before starting cART have an increased virological failure risk. On the contrary, in their meta-analysis, Skowron *et al.*[31] found that baseline CD4^+^ cell count was a critical predictor of virologic suppression. More recently, Stirrup *et al.*[32] evidenced how a lower CD4^+^ cell baseline is a predictor of virological failure with drug resistance in the UK CHIC study. Some of these discrepancies are likely to be because of the study design. For example, the CD4^+^ cell count measured at the time of viral suppression (like in our analysis) is likely to be a predictor of subsequent risk of viral rebound as it reflects adherence to ART received before baseline.

Previous virological failures remained a significant predictor of losing ‘U status’ by multivariable model in our analysis. Moreover, PWH, who never experienced a virological failure, spent significantly lower PDFU with viral load above 200 copies/ml (2.9%) when compared with those who have had one to three (4.4%) and more than three previous episodes of virological failure (6.3%). These results were in accordance with previous observations from a number of other studies. Rusconi *et al.* showed how the risk of virological failures was decreasing in the last year; despite that, the risk associated with viral rebound according to the previous history of virological failure remained constant over time [33]. In the same study, other factors independently associated with a lower risk of virological failure were Italian origin, a longer history of virological suppression, and maximum level of education achieved (those with a university degree having the lowest risk). Similar results were obtained by Reekie *et al.*[34], who showed that the rate of virological failure was significantly related to the number of viral rebounds the patient had experienced in the EUROSIDA cohort.

Our study has some limitations. Firstly, it can be argued that the definition of losing the ‘U status’ based on 10% of PDFU spent with a viral load above 200 copies/ml is arbitrary and highly dependent on the duration of follow-up. Indeed the longer the follow-up, the greater the chance to reach this endpoint. However, when we used the alternative endpoint of 5% of PDFU and of more than 3 months with a viral load greater than 200 copies/ml, the results were similar.

Secondly, the frequency of viral load measurements is likely to vary from patient to patient, which could have affected the calculation of the PDFU. Specifically, PDFU could be more accurate for people with more frequent measures, although as we used a ‘most recent value carried forward’ approach, we believe that any bias is probably negligible. Also, we performed a sensitivity analysis in a subset of the cohort of participants whose HIV-RNA was monitored at least twice/year, and the results were again similar. If anything, the estimate of 3.1% of PDFU spent in the ‘U status’ is an under-estimate of what happened to the average person who is stably in care in our clinics.

Thirdly, considering that our study is a retrospective analysis and no data about HIV-negative partners was available, we could not assess whether HIV sexual transmissions in PWH who have lost the ‘U status’ have occurred. Similarly, we identified factors associated with losing the ‘U status’, not the risk of HIV sexual transmission, and we are assuming that by reducing the proportion of people leaving the ‘U status’, we could achieve less new infections.

Last but not least, adherence is a key unmeasured confounder in all these analyses, and failing to control for adherence could have biased the magnitude and significance of some of the associations described.

With evidence coming from both clinical trials and observational studies supporting these efforts, clinicians should correctly communicate the U=U prevention message to the PWH [11–14]. However, a recent international survey found that only 77% of infectious disease specialists and 42% of primary care physicians communicated the message to patients when informing them of their undetectable viral load level [35].

In conclusion, our data show an ecological correlation between the introduction of the U=U campaign and a scenario of unfrequent episodes of viral load greater than 200 copies/ml over a long period of time with a further trend toward a decrease after the launch of the campaign. U=U is an essential but straightforward campaign founded on scientific evidence. It has already successfully influenced public opinion, reducing the stigma in PWH and consequently improving their quality of life.

Nevertheless, our and previous studies also show that more efforts need to be made to encourage clinicians to introduce this message during routine visits. Our data are useful to identify subpopulations (e.g. women and foreign-born) who may particularly benefit from targeted interventions.

## Acknowledgements

Icona Foundation Study Group: BOARD OF DIRECTORS: A. d’Arminio Monforte (President), A. Antinori (Vice-President), M. Andreoni, A. Castagna, F. Castelli, R. Cauda, G. Di Perri, M. Galli, R. Iardino, G. Ippolito, A. Lazzarin, G.C. Marchetti, G. Rezza, F. von Schloesser, P. Viale.

SCIENTIFIC SECRETARY: A. d’Arminio Monforte, A. Antinori, A. Castagna, F. Ceccherini-Silberstein, A. Cozzi-Lepri, E. Girardi, A. Gori, S. Lo Caputo, F. Maggiolo, C. Mussini, M. Puoti, C.F. Perno. STEERING COMMITTEE: A. Antinori, F. Bai, A. Bandera, S. Bonora, M. Borderi, A. Calcagno, M.R. Capobianchi, A. Castagna, F. Ceccherini-Silberstein, S. Cicalini, A. Cingolani, P. Cinque, A. Cozzi-Lepri, A. d’Arminio Monforte, A. Di Biagio, R. Gagliardini, E. Girardi, N. Gianotti, A. Gori, G. Guaraldi, G. Lapadula, M. Lichtner, A. Lai, S. Lo Caputo, G. Madeddu, F. Maggiolo, G. Marchetti, C. Mussini, S. Nozza, C.F. Perno, S. Piconi, C. Pinnetti, M. Puoti, E. Quiros Roldan, R. Rossotti, S. Rusconi, M.M. Santoro, A. Saracino, L. Sarmati, V. Spagnuolo, V. Svicher, L. Taramasso. STATISTICAL AND MONITORING TEAM: A. Cozzi-Lepri, I. Fanti, P. Lorenzini, A. Rodano’, M. Macchia, A. Tavelli. COMMUNITY ADVISORY BOARD: A. Bove, A. Camposeragna, M. Errico, M. Manfredini, A. Perziano, V. Calvino. BIOLOGICAL BANK INMI: F. Carletti, S. Carrara, A. Di Caro, S. Graziano, F. Petroni, G. Prota, S. Truffa. PARTICIPATING PHYSICIANS AND CENTERS: Italy -- A. Giacometti, A. Costantini, V. Barocci (Ancona); G. Angarano, L. Monno, E. Milano (Bari); F. Maggiolo, C. Suardi (Bergamo); P. Viale, V. Donati, G. Verucchi (Bologna); F. Castelnuovo, C. Minardi, E. Quiros Roldan (Brescia); B. Menzaghi, C. Abeli (Busto Arsizio); L. Chessa, F. Pes (Cagliarti); B. Cacopardo, B. Celesia (Catania); J. Vecchiet, K. Falasca (Chieti); A. Pan, S. Lorenzotti (Cremona); L. Sighinolfi, D. Segala (Ferrara); P. Blanc, F. Vichi (Firenze); E. Pontali, M. Bassetti, A. Alessandrini, N. Bobbio, G. Mazzarello (Genova); M. Lichtner, L. Fondaco, (Latina); S Piconi, C. Molteni (Lecco); A. Chiodera, P. Milini (Macerata); G. Nunnari, G. Pellicanò (Messina); A. d’Arminio Monforte, M. Galli, A. Lazzarin, G. Rizzardini, M. Puoti, A. Castagna, F Gelpi, M.C. Moioli, R. Piolini, D. Bernacchia, A. Poli, C. Tincati, (Milano); C. Mussini, C. Puzzolante (Modena); P Bonfanti, G. Lapadula (Monza); V. Sangiovanni, G Di Filippo, V. Esposito, G. Di Flumeri, I. Gentile, V. Rizzo (Napoli); A.M. Cattelan, S. Marinello (Padova); A. Cascio, M. Trizzino (Palermo); D. Francisci, E. Schiaroli (Perugia); G. Parruti, F. Sozio (Pescara); C. Lazzaretti, R. Corsini (Reggio Emilia); M. Andreoni, A. Antinori, R. Cauda, V. Vullo, V Mazzotta, S. Lamonica, M. Capozzi, A. Mondi, A. Cingolani, M. Rivano Capparuccia, G. Iaiani, A. Latini, G. Onnelli, M.M. Plazzi, G. De Girolamo, A. Vergori (Roma); M. Cecchetto, F. Viviani (Rovigo); G. Madeddu, A. De Vito(Sassari); M Fabbiani, F. Montagnani (Siena); A. Franco, R. Fontana Del Vecchio (Siracusa); C. Di Giuli (Terni); G Calleri, G. Di Perri, S. Bonora, G.C. Orofino, M. Sciandra (Torino); A. Londero (Udine); V. Manfrin, G. Battagin (Vicenza); G. Starnini, A. Ialungo (Viterbo).

Authors’ contribution: G.M., A.D.V., A.C.L., A.D.M., A.A., and E.G. conceived the study. A.C.L. performed the statistical analyses. A.D.V., A.C., F.M., C.F.P., R.G., G.M., and A.S. contributed to the patients’ enrolment and data collection. G.M., A.D., A.C.L., A.D.M., A.A., and E.G. wrote the first version of the manuscript. All authors reviewed the manuscript, provided critical scientific revisions, and approved the final version of the manuscript.

### Conflicts of interest

G.M. reports personal fees from Gilead Sciences, personal fees from Janssen, personal fees from Merck Sharp and Dohme, personal fees from ViiV, outside the submitted work; F.M. reports grants and personal fees from ViiV, grants and personal fees from Gilead, personal fees from MSD, grants and personal fees from Jannsen, outside the submitted work; R.G. reports personal fees from ViiV, personal fees from Merck, grants from Gilead, outside the submitted work; A.S. reports grants from GILEAD, outside the submitted work; A.D.M. reports grants, personal fees, and nonfinancial support from Gilead Sciences, personal fees from Janssen-Cilag, personal fees from Merck, personal fees and nonfinancial support from ViiV Healthcare, outside the submitted work; A.A. reports grants, personal fees, and nonfinancial support from Gilead Sciences, grants and personal fees from Janssen-Cilag, personal fees from Merck, grants, personal fees, and nonfinancial support from ViiV Healthcare, outside the submitted work; E.G. reports personal fees from ViiV, personal fees from Gilead, grants from Gilead, grants from Mylan, personal fees from Mylan, personal fees from Angelini, outside the submitted work; A.D.V., A.C.L., A.C., C.F.P., and G.M. have nothing to disclose.

## Supplementary Material

Supplemental Digital Content
