# Supplementary material for: Time spent with HIV-RNA ≤ 200 copies/ml in a cohort of people with HIV during the U=U era
Source: AIDS. 2021 Feb 9;35(7):1103–12. doi: 10.1097/QAD.0000000000002825 (PMC9904439; doi:10.1097/QAD.0000000000002825)
Supplement: Supplemental Digital Content [file aids-35-1103-s001.docx]

****Supplemental Table 1**. Odds Ratio of U=U status (≤3 months with VL>200) from fitting a logistic regression**

| **Logistic regression estimates of factors associated with U=U status** | | | | | | |
| --- | --- | --- | --- | --- | --- | --- |
|  | | **Unadjusted** | | **Adjusted^*^** | |  |
| **Factor** | **Odds ratio (95% CI)** | | **p-value** | **Odds ratio (95% CI)** | **p-value** | **Type III p-value** |
| ***Gender*** | |  |  |  |  |  |
| Female vs. Male | | 0.49 (0.41, 0.57) | <.001 | 0.73 (0.58, 0.92) | 0.007 |  |
| ***Mode of HIV Transmission*** | |  |  |  |  | <.001 |
| MSM vs. PWID | | 3.84 (3.06, 4.83) | <.001 | 2.27 (1.70, 3.03) | <.001 |  |
| Heterosexual vs. PWID | | 1.68 (1.36, 2.07) | <.001 | 1.36 (1.05, 1.76) | 0.020 |  |
| Other/Unknown vs PWID | | 1.92 (1.37, 2.69) | <.001 | 1.21 (0.81, 1.83) | 0.355 |  |
| ***Employment, n(%)*** | |  |  |  |  | <.001 |
| Employed vs. Unemployed | | 1.69 (1.37, 2.09) | <.001 | 1.32 (1.04, 1.67) | 0.021 |  |
| Occasional vs. Unemployed | | 0.92 (0.62, 1.35) | 0.656 | 1.09 (0.72, 1.65) | 0.685 |  |
| House work vs. Unemployed | | 0.82 (0.55, 1.20) | 0.305 | 1.22 (0.78, 1.90) | 0.388 |  |
| ***CD4 count, cells/mmc*** | |  |  |  |  |  |
| per 100 higher | | 1.04 (1.01, 1.07) | 0.003 | 1.03 (0.99, 1.06) | 0.105 |  |
| ***CD8 count, cells/mmc*** | |  |  |  |  |  |
| per 100 higher | | 0.99 (0.98, 1.01) | 0.473 | 0.98 (0.96, 0.99) | 0.002 |  |
| ***Previous VF*** | |  |  |  |  | <.001 |
| 1-3 vs. 0 | | 0.37 (0.29, 0.48) | <.001 | 0.54 (0.38, 0.77) | <.001 |  |
| 3+ vs. 0 | | 0.24 (0.18, 0.30) | <.001 | 0.44 (0.30, 0.64) | <.001 |  |
| ^*^Multivariable model includes all variables selected by backward selection that were retained with a p-value less than 0.3 level. Also adjusted for age, AIDS diagnosis, HBsAg/HCV status, duration of ART, anchor drug used, geographical region, diabetes, smoking, use of statins/lowering blood pressure drugs, glucose and prior STDs. PWID: people who inject drugs; MSM: men who have sex with men. | | | | | | |

**Supplemental Figure 1. Spaghetti plot of individual participants’ HIV-RNA trajectory (selected subset of people with ≥2 episodes of sustained HIV-RNA>200 copies/mL).**

**Supplemental Figure 2. Adjusted OR from fitting a logistic regression model restricted to the subset of participants with ≥2 HIV-RNA measures/year**
